# Canonical or non‐canonical, all aspects of G protein‐coupled receptor kinase 2 in heart failure

**DOI:** 10.1111/apha.70010

**Published:** 2025-02-17

**Authors:** Abdullah Kaplan, Lana El‐Samadi, Rana Zahreddine, Ghadir Amin, George W. Booz, Fouad A. Zouein

**Affiliations:** ^1^ Department of Pharmacology and Toxicology American University of Beirut Faculty of Medicine Beirut Lebanon; ^2^ The Cardiovascular, Renal, and Metabolic Diseases Research Center of Excellence American University of Beirut Medical Center Beirut Lebanon; ^3^ Cardiology Clinic Kemer Public Hospital Antalya Turkey; ^4^ Department of Pharmacology and Toxicology, School of Medicine University of Mississippi Medical Center Jackson Mississippi USA

**Keywords:** G protein‐coupled receptors, GRK2, signal transduction, β‐Adrenergic receptor kinase 1, β‐Adrenergic receptors, β‐Arrestins

## Abstract

G protein‐coupled receptor kinase 2 (GRK2) with its multidomain structure performs various crucial cellular functions under both normal and pathological conditions. Overexpression of GRK2 is linked to cardiovascular diseases, and its inhibition or deletion has been shown to be protective. The functions of GRK2 extend beyond G protein‐coupled receptor (GPCR) signaling, influencing non‐GPCR substrates as well. Increased GRK2 in heart failure (HF) initially may be protective but ultimately leads to maladaptive effects such as GPCR desensitization, insulin resistance, and apoptosis. The multifunctional nature of GRK2, including its action in hypertrophic gene expression, insulin signaling, and cardiac fibrosis, highlights its complex role in HF pathogenesis. Additionally, GRK2 is involved in mitochondrial biogenesis and lipid metabolism. GRK2 also regulates epinephrine secretion from the adrenal gland and its increase in circulating lymphocytes can be used to monitor HF status. Overall, GRK2 is a multifaceted protein with significant implications for HF and the regulation of GRK2 is crucial for understanding and treating cardiovascular diseases.

## 1 INTRODUCTION

G protein‐coupled receptors (GPCRs) are the largest family of transmembrane proteins and are involved in many cellular activities in response to hormones and neurotransmitters.^1^, ^2^ In the heart, β‐adrenergic receptors (βARs) are a well‐known type of GPCRs, regulating a variety of functions.^2^, ^3^ G protein‐coupled receptor kinase (GRKs) and β‐arrestin (βarr) act as key regulators of GPCR signaling^4^ with GRK2 being the predominant GRK family isoform present in the heart.^5^, ^6^ For myocardial signaling and function, either in physiological or pathological conditions, GRK2 plays a critical role and therefore is a potential target for heart failure (HF) therapy.^7^ While GRK2 is indispensable for heart development in the embryo, and GRK2‐knockout mice die at an early stage of embryonic development due to cardiac hypoplasia,^8^, ^9^ overexpression of GRK2 is associated with a variety of pathologies such as HF, hypertension, diabetes, inflammation, and osteoporosis.^9^ Conversely, either inhibition or deletion of GRK2 before or after induction of cardiac injury is protective.^10^, ^11^ Besides mediating canonical GPCR signaling in the cytoplasm, GRK2 can phosphorylate many non‐GPCR substrates and is involved in various pathologic conditions.^12^, ^13^, ^14^

βarr is a multifunctional scaffold protein functioning in the internalization and desensitization of GPCRs.^13^ Upon activation of βARs, GRK2 is recruited to the plasma membrane and phosphorylates the receptor, and subsequently βarr is recruited and binds to the receptor, resulting in receptor desensitization and clathrin‐mediated receptor internalization with either its recycling or degradation.^4^, ^11^

HF affects an estimated 64 million people worldwide and its prevalence is expected to increase due to the aging of the population. Most projections for the US suggest that HF prevalence will increase by about 46% from 2012 to 2030.^15^ A meta‐analysis of echocardiographic screening studies in the general population from developed countries reported that HF had a median prevalence rate of 11.8% in people 60 years or older.^15^, ^16^ The ECHOES study with patients at a mean age of 64 reported a 10‐year survival rate of 26.7% for those with HF, compared to 75% for participants without HF.^15^, ^17^

HF is associated with various activated neuroendocrine systems, including the renin‐angiotensin‐aldosterone system, the arginine vasopressin system and the sympathetic nervous system. Sustained activation of these systems leads to the perpetuation of the pathophysiology and worsening of the clinical symptoms.^18^ High levels of catecholamines impair myocardial metabolism and increase oxygen demand, which promotes necrosis and inflammation and subsequently leads to interstitial fibrosis. Alterations in myocardial glucose and lipid metabolism along with the development of insulin resistance and mitochondrial dysfunction are characteristics of HF.^14^

**FIGURE 1:**
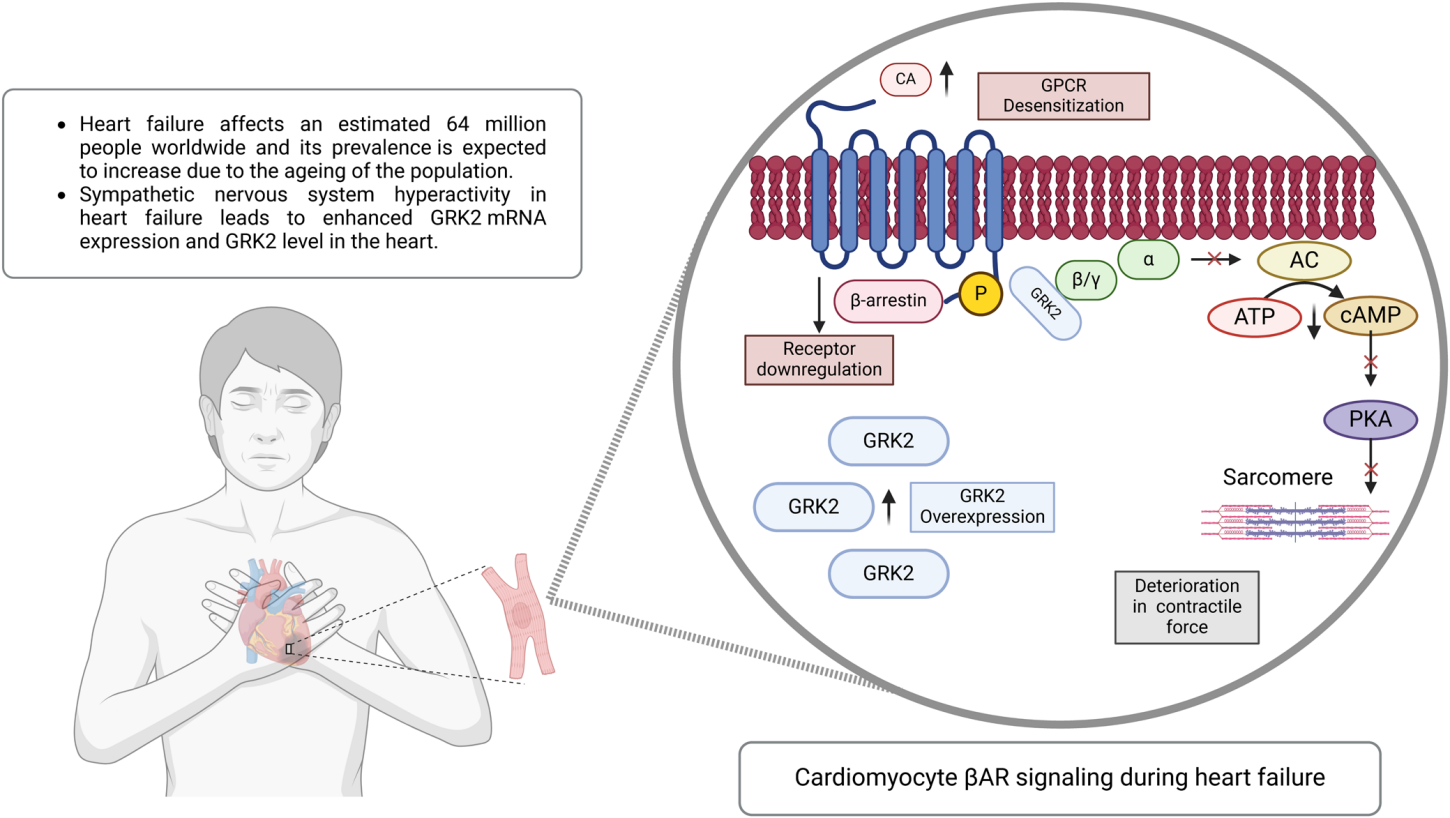
The Action of GRK2 on Cardiac ΒAR Signaling During Sympathetic Nervous System Hyperactivity in Heart Failure. Upon receptor activation, GRK2 translocates to the membrane and phosphorylates the receptor, triggering G protein uncoupling. β‐arrestins then bind to the phosphorylated receptor, blocking G protein activation, promoting receptor internalization and downregulation, and initiating new signaling pathways. During heart failure, increased catecholamines elevate GRK2 levels, which cause chronic desensitization of β‐adrenergic receptor and pathological effects, contributing to lower cardiac contractile function and heart failure progression. GRK2, G‐protein‐coupled receptor kinase 2; GPCR, G‐protein‐coupled receptor; AC, adenylate cyclase; CA, catecholamines; cAMP, cyclic adenosine monophosphate; ATP, adenosine triphosphate; PKA, protein kinase A; βAR, β‐adrenergic receptor.

Sympathetic nervous system hyperactivity in HF leads to enhanced GRK2 mRNA expression and GRK2 level in the heart. Initially, enhanced GRK2 levels may be protective for myocardial tissue by counterbalancing beta‐adrenergic overdrive, but persistent GRK2 overactivity is maladaptive and paves the way for HF by leading to GPCR desensitization and downregulation, insulin resistance, mitochondrial dysfunction, and apoptosis (Figure 1).^19^, ^20^, ^21^

## 2 GRK FAMILY

The GRK family consists of seven members (GRK1‐7) and based on gene structure and sequence homology, is divided into three subgroups: the visual or rhodopsin‐kinase subfamily (GRK1 and GRK7), the βAR kinase subfamily (GRK2 and GRK3), and the GRK4 subfamily (GRK4, GRK5, GRK6).^22^, ^23^ These seven GRKs regulate over 800 GPCRs.^23^ GRK is a serine/threonine kinase and among the GRK family, GRK2 and GRK5 are expressed in almost all cardiac cells, whereas GRK3 is primarily detected in cardiac myocytes.^5^, ^6^, ^24^, ^25^ GRK consists of multiple domains, and the expression patterns of these domains vary depending on the GRK subtype and cell type.^26^ GRK2 has 5 domains: α‐N‐terminal domain, N‐terminal regulator of G‐protein signaling (RGS) homology domain (RH), central catalytic domain, C‐terminal domain, and a pleckstrin homology domain (PH).^23^, ^26^, ^27^

Upon agonist stimulation, β_1_AR is phosphorylated by GRKs and subsequently associates with βarr, leading to receptor desensitization and internalization. The activity of GRKs is enhanced after stress/injury and pathologic upregulation of these molecules gives rise to excessive signal uncoupling and receptor desensitization which promote pathogenesis.^13^ Not only GPCRs but also non‐GPCR substrates are known to be phosphorylated by GRKs.^12^ GRK2 can bind structural proteins such as β‐tubulin and HDAC6, and interact with heat shock protein 90 (HSP90), aldosterone–mineralocorticoid receptor system, and insulin receptor substrate 1 (IRS1).^13^, ^28^ Hence, GRK2 can play a role in cytoskeletal functions, oxidative stress, glucose uptake, and insulin resistance,^13^ and is involved in several cardiovascular pathologies such as hypertension and hypertrophic cardiomyopathy, metabolic syndrome, type 2 diabetes, and nonalcoholic fatty liver disease.^14^, ^29^

GRK2 exerts various functions through distinct functional domains such as the central catalytic domain and amino and carboxyl‐terminal regulatory domains.^30^ The amino terminal regulator of G protein signaling or RGS RH can selectively interact with G proteins and suppress pathological cardiac hypertrophy.^30^, ^31^ Most GRK2 inhibitors, such as paroxetine, inhibit the kinase activity of GRK2 but leave this cardioprotective RGS domain of GRK2 intact.^10^

GRK2 is one member of the A, G, and C family (AGC family) of kinases that play a vital role in many cell activities with aberrant actions involved in several diseases.^32^, ^33^, ^34^ Since AGC kinases have high sequence and structural similarity at the kinase domain, the inhibition of GRK2 may cause inhibition of other AGC kinases.^34^, ^35^ Moreover, cross activity between GRK2 and other AGC family of kinases such as GRK1, GRK3, and protein kinase A (PKA) have been shown.^34^, ^35^, ^36^, ^37^ Therefore, selective inhibition of GRK2 is essential to avoid potential unfavorable effects resulting from the inhibition of other AGC kinases (Table 1).^34^

**TABLE 1:** Pharmacological modulators of βAR Kinase subfamily, GRK2.

Drug	Function
Isoproterenol	Non‐selective β agonist
βARKct peptide	GRK2 inhibitor, binds to Gβγ subunit and prevents GRK2 from being recruited to the cell membrane
Paroxetine	GRK2 inhibitor, a selective serotonin reuptake inhibitor
Prostaglandin E2	Suppresses TGF‐β1‐GRK2 interaction in cardiomyocytes and cardiac fibroblasts
Gallein	Gβγ subunit inhibitor
RAF kinase inhibitor protein	Inhibits GRK2 by interacting with the N‐terminal domain of GRK2, inhibits pro‐survival RAF1‐MAPK pathway and attenuates βAR desensitization
Proteasome inhibitors	Prevents early GRK2/Pin1/Akt degradation
Calpain inhibitors	Prevents early GRK2/Pin1/Akt degradation
Adenoviral‐βARKct	Peptide inhibitor of betaARK1
KRX‐C7 peptide	Selective inhibitor of GRK2, increases insulin sensitivity
Aldosterone	Upregulates GRK2, weakens insulin signaling, enhances the negative phosphorylation of IRS1, and reduces Akt activity
CMPD101	GRK2 inhibitor
GRK2 siRNA	Molecular suppressor of GRK2 expression
Methyl 5‐[2‐(5‐nitro‐2‐furyl)vinyl]‐2‐furoate	Chemical inhibitor of GRK2, selective inhibitor of kinase activity of GRK2

GRKs, in particular GRK2 isoform, participate in many cellular and physiological processes by interacting with a variety of non‐GPCR proteins. GRK2 multifunctionality is related to its multidomain structure as well its expression levels, activity, and localization within the cell.^26^ Non‐GPCR substrates and cellular interactors include ligases and chaperones (Cul4A‐DDB1‐Gβ, Nedd4‐2, Mdm2, Hsp90), receptors and membrane proteins (β‐ENaC, EGFR, PDGFR, IGF1R), cytoplasmic kinases (p38, Akt, AMPK, PKC, ERK, Src, PI3Kγ, MEK, MST1), signaling switchers (Gαq, RhoA, Epac1, RalA), signal transducers (HDAC6, Pin1, RKIP, eNOS, APC, Smad, IκBα, PDEγ, IRS1, GIT), cytoskeletal proteins and regulators, intracellular protein transport, organelle maintenance (β‐tubulin, α‐actinin, clathrin, Ezrin/radixin, α and β‐synuclein, caveolin, mitofusin), and transcription factors (Dream, Period1/2).^26^

## 3 G PROTEINS

G proteins convert extracellular stimuli into intracellular responses upon coupling to GPCRs.^38^ G proteins consist of *alpha* (α), *beta* (β) and *gamma* (γ) subunits and are subdivided into four families: Gα_i_, Gα_12/13_, Gα_q_, and Gα_s_.^39^, ^40^ Gα_s_ stimulates adenylyl cyclase, increasing cyclic adenosine monophosphate (cAMP), while Gα_i_ suppresses adenylyl cyclase activity, resulting in decreased intracellular cAMP levels.^40^ In the resting state, the Gα subunit, with bound guanosine diphosphate (GDP), forms a heterotrimeric structure with the Gβγ subunit dimer. Following extracellular stimulation, a conformational change occurs in the GPCR resulting in G protein activation. Thus, GDP is exchanged for guanosine triphosphate (GTP) and subsequently, the Gα subunit dissociates from the Gβγ dimer and activates downstream effector proteins.^38^ Activated βAR exposes a site for Gαs protein to bind, which in turn leads to the activation of adenylyl cyclase, producing cAMP.^41^, ^42^ At the end, the α subunit hydrolyzes GTP to GDP, which leads back to Gαβγ heterotrimer formation and signal termination.^38^ Sustained GPCR activation causes cytotoxicity and to avoid this, β_1_AR is desensitized and endocytosed over time. This process starts with the phosphorylation of activated β_1_AR, with βarr in the cell binding to phosphorylated β_1_AR, which in turn blocks binding between Gαs protein and β_1_AR and promotes the endocytosis of β_1_AR.^43^

## 4 Β‐ARRESTIN

βarr1 and 2, among the four‐member βarr family, are well‐known scaffold and multifunctional intracellular proteins that regulate the activity of a very large number of cellular signaling pathways.^13^, ^44^ Arrestins exist in four distinct forms, according to evidence now available: free monomers, free oligomers, GPCR‐bound, and microtubule‐bound.^44^, ^45^ Arrestins are elongated proteins made up of two regions, commonly referred to as the N‐ and C‐domains.^44^ The traditional signaling paradigm states that arrestins are drawn to G protein‐bound GPCRs following GRK phosphorylation of the receptor's C‐terminal tail.^46^ However, recent investigation revealed that βarr trafficking could be activated without a phosphorylated GPCR tail.^47^

Structural investigations have discovered two major GPCR/arrestin interaction sites. The arrestin finger‐loop region enters into the cytoplasmic cavity created by the GPCR transmembrane core, while the arrestin N‐domain attaches to phosphorylated areas of the receptor. Furthermore, loops in arrestin's C‐edge have been shown in recent research to serve as a membrane anchor, improving the stability of GPCR/arrestin complexes.^44^ Using atomic‐level simulations, researchers discovered that both the transmembrane core and cytoplasmic tail of GPCRs can independently activate arrestin by binding to different surfaces.^48^ When a receptor is phosphorylated, βarrs are drawn to the receptor by binding its phosphorylated tail. Further interactions with the intracellular loops and core of the activated receptors allow for various conformational states of the arrestin‐receptor complexes. This recruitment of βarrs physically obstructs the receptor's interactions with the Gα subunit, leading to receptor desensitization by limiting additional signaling from G‐proteins.^49^ Notably, arrestin can remain active without the receptor when its C‐terminal tail is disengaged, explaining its prolonged activity.^48^ In order to effectively block G protein signaling, βarrs not only physically bind to active GPCRs to prevent G protein coupling, but also start endocytosis and kinase activation. The fact that βarrs modulate/mediate a wide range of cellular activities through either GPCR‐dependent or ‐independent pathways is now well established.^48^

Over the past two decades, research has shown that some GPCR signaling can occur independently of G proteins, with βarrs acting as signal transducers and scaffolds for signaling complexes, particularly in the activation of the extracellular signal‐ regulated kinase 1/2 (ERK1/2) cascade. However, recent genome‐editing studies revealed that while G proteins are essential for ERK activation, βarrs are not. Despite this, βarrs are still recruited to activated GPCRs even without active G proteins. This G protein‐independent arrestin activation allows receptor internalization but is insufficient to trigger ERK signaling.^46^, ^50^, ^51^

The knockdown of arrestins affects the amplitude and timing of ERK activation in different ways, depending on the GPCR type. For example, for βARs, arrestin depletion increases ERK activation for β_2_ARs but decreases it for β_1_ARs. This shows that arrestins play a desensitizing role for β_2_ARs, while acting as signaling scaffolds for β_1_ receptors. However, even with arrestin removal, ERK signaling is not fully repressed in any case, indicating that arrestins modulate but are not essential for ERK activation.^46^, ^47^, ^50^ Available evidence suggests that arrestins function as rheostats in the context of ERK signaling, controlling the location, duration, and intensity of intracellular and/or cell surface ERK stimulation rather than directly activating ERK.^46^

While most drugs targeting GPCRs are traditionally thought to affect all signaling pathways equally, it was recognized decades ago that some drugs could specifically target certain receptor‐linked systems. Over the past two decades, “functionally selective” or “biased” agonists have been identified. These ligands can selectively activate certain pathways (e.g., G proteins) while blocking others (e.g., arrestins), offering a more targeted approach compared to traditional “balanced” agonists.^52^, ^53^, ^54^ Biased responses can result from preferential signaling through G proteins or βarrs, which can be driven by biased ligands, biased receptors, or system bias.^52^

## 5 ADRENERGIC RECEPTORS

Adrenergic receptors, members of the GPCR superfamily, are divided into 3 types and 9 subtypes: 3 α_1_AR subtypes (α_1A_, α_1B_, α_1D_), 3 α_2_AR subtypes (α_2A_, α_2B_, α_2C_), and 3 βAR receptors (β_1_, β_2_, β_3_).^55^, ^56^ All 3 types of βARs are expressed in the human heart.^56^, ^57^ In normal heart β_1_AR is the most common subtype, accounting for 90% of all βAR density, followed by β_2_AR (15%–18%) and β_3_AR (2%–3%).^56^, ^58^ Using isolated mouse cardiomyocytes, β_1_ and α_1_B were revealed as the dominant adrenergic receptors and present in all cells, whereas only about 5% of cardiomyocytes expressed β_2_‐ and β_3_ARs, both of which were abundantly detected in nonmyocytes. The α_1A_ receptor was detected at high levels in only 20% of cardiomyocytes.^59^

While β_2_AR is mostly concentrated in transverse tubules (T‐tubules) and lipid rafts, β_1_AR is widely distributed across the cell, providing more diffuse cytosolic cAMP signal.^1^, ^60^, ^61^ Unlike β_1_‐AR, β_2_AR can couple to Gα_i_ proteins in addition to Gα_s_ proteins.^41^, ^43^, ^62^ By binding to the Gαi protein, β_2_AR blocks β_1_AR signaling, exerting cardioprotective effects.^43^, ^62^ Furthermore, β_2_AR signaling can convert β_1_AR signaling from global to local mode by targeting the C terminus of β_1_ARs, protecting the heart from the cytotoxic effects of circulating catecholamine.^63^ The cardioprotective effects of β_2_AR appear to be highly dependent on its expression level and higher expression levels lead to enhanced myocardial fibrosis and cardiomyopathy.^64^ β_3_AR counteracts β_1_AR and β_2_AR through activation of an NOS pathway to balance the effects of catecholamines on the heart, preventing myocardial dysfunction.^65^

In HF, cardiomyocytes are exposed to high levels of catecholamines, derived from cardiac sympathetic nerve endings (norepinephrine) and the adrenal medulla (epinephrine).^56^ As in cardiac tissue, GRK2 is upregulated in the adrenal gland in HF.^7^, ^66^, ^67^ The α_2_ARs, involved in catecholamine secretion, are uncoupled and downregulated in the adrenal gland by GRK2 in HF (Figure 3).^7^ Agonism of α_2_AR may provide protection to the heart from oxidative damage. This was recently shown in the H9c2 rat cardiomyoblast cell line, where the α_2_AR agonist dexmedetomidine notably decreased oxidative stress and apoptosis caused by hydrogen peroxide (H_2_O_2_).^68^, ^69^ However, nicotine can hinder the capacity of α_2_ARs to deliver antioxidant and anti‐apoptotic protection for the heart. The underlying mechanism seems to involve the upregulation of GRK2, which greatly disrupts α_2_AR‐mediated antioxidant signaling in H9c2 cardiomyocytes.^68^

**FIGURE 2:**
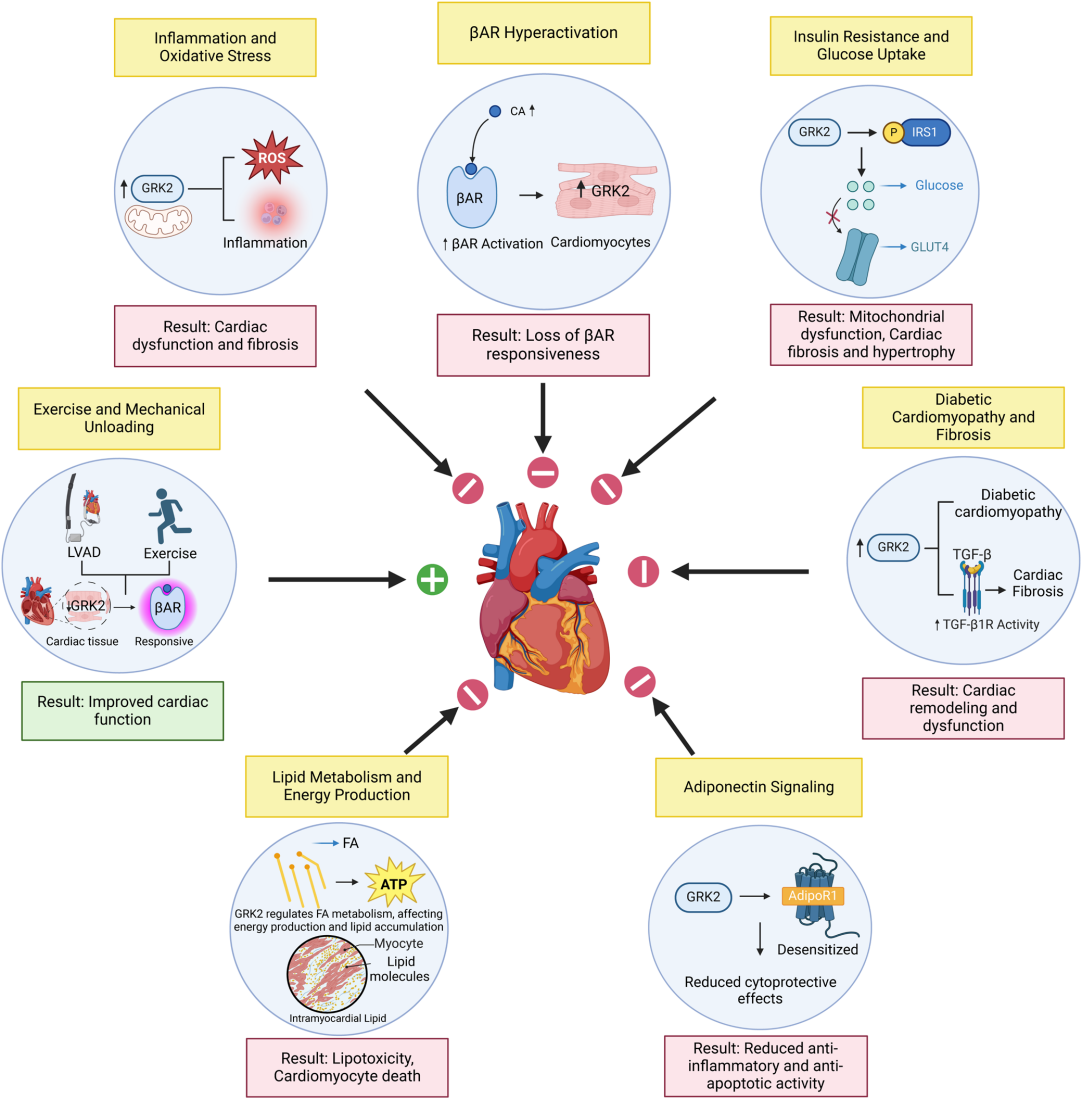
Mechanisms Affecting Heart Function via GRK2 in Cardiovascular Pathophysiology. The figure illustrates the various pathways through which GRK2 impacts heart function, particularly in the context of cardiovascular diseases. βAR hyperactivation, indicated by increased GRK2 in cardiomyocytes due to high catecholamine levels, results in reduced βAR responsiveness. Insulin resistance is mediated by GRK2's phosphorylation of IRS1, reducing glucose uptake and causing mitochondrial dysfunction, cardiac fibrosis, and hypertrophy. In diabetic cardiomyopathy, high GRK2 levels are linked to early‐stage diabetic cardiomyopathy and subsequent cardiac dysfunction. Increased GRK2 levels enhance TGF‐β1 activity, leading to increased fibrosis. Additionally, GRK2 desensitizes AdipoR1, reducing the receptor's cytoprotective effects and leading to diminished anti‐inflammatory and anti‐apoptotic activity. GRK2's effects on fatty acid metabolism, lead to lipotoxicity and cardiomyocyte death. Conversely, exercise and mechanical unloading through devices like LVAD reduce GRK2 levels, improving βAR responsiveness and cardiac function. Increased GRK2 activity also correlates with oxidative stress and inflammation, contributing to cardiac dysfunction and fibrosis. βAR, β‐adrenergic receptor; CA, catecholamines; GRK2, G‐protein‐coupled receptor kinase 2; IRS‐1, insulin receptor substrate 1; GLUT 4, glucose transporter type 4; TGF‐β1, transforming growth factor beta 1; AdipoR1, adiponectin receptor 1; FA, fatty acids; ATP, adenosine triphosphate; LVAD, left ventricular assist device; ROS, reactive oxygen species.

In early stage of HF, acute stimulation of cardiac βARs by epinephrine and norepinephrine enhance intracellular cAMP production and PKA activation, both of which have a critical role in the regulation of contraction and relaxation of cardiac myocytes.^1^, ^70^ Indeed, sustained stimulation of cardiac βARs by excessive sympathetic nervous system activity can trigger adverse effects, including disproportionate increases in energy consumption, apoptosis, fibrosis, cardiomyocyte hypertrophy, and arrhythmia,^1^, ^71^ and the continual deterioration in myocyte contractile force and adverse cardiac remodeling (Figure 2).^7^ To protect from the toxic effects of circulating catecholamines, β_1_AR density decreases.^1^, ^72^ Moreover, the β_2_AR uncouples from Gαs, resulting in less cAMP production, although its density does not alter significantly in HF. Increased myocardial Gαi expression in HF provides further contribution of β_2_AR signaling in the context of protection.^1^, ^62^, ^73^ On the other side, in the chronic stage, β_2_AR may redistribute from T tubules to the surface of cardiomyocytes, like β_1_AR, leading to diffuse cAMP signaling, and loss of cardioprotective activity.^1^, ^60^, ^61^

β_1_AR endocytosis is one of the crucial mechanisms for terminating signal transduction driven by a high level of chronic catecholamine exposure.^43^ However, this mechanism does not work properly in the presence of autoantibodies against β_1_AR, which circulate in the sera of 30%–40% of HF patients.^43^, ^74^, ^75^ The reason might be ineffective GRK2 activation by β_2_AR under the influence of the autoantibodies.^43^ Studies suggest that the autoantibodies against β_1_AR can restrict β_1_AR endocytosis and render the receptor persistently stimulated by the autoantibodies, leading to overactivation of β_1_AR downstream signaling and myocardial injury.^43^, ^76^ The β_2_AR, via the Gαi signaling pathway, confers cardioprotection by promoting the endocytosis of β1AR and thereby terminating its signaling activity.^43^

These findings are distinct from the previous reports to some extent. It is well‐established that downregulation of adrenergic receptors is linked to the hyperadrenergic state in HF.^77^ Likewise, the effect of β_1_AR autoantibodies on βARs in neonatal rat cardiomyocytes displayed similar downregulatory results observed in failing human hearts.^78^ In a human‐analogous rat model of HF caused by antibody, a cyclic peptide mimicking the second extracellular loop of the β_1_AR restored the downregulated receptors on the cell surface as a result of antibody scavenging and decreased production. Additionally, increases in the expression of GRK2 and GRK5 were nearly completely reversed. It is necessary to resolve several discrepancies pertaining to β_1_AR autoantibodies. The prevalence of β_1_AR autoantibodies in HF is uncertain because there is no widely accessible, standardized, validated, or affordable diagnostic test for these antibodies.^77^, ^79^, ^80^ Not all identified β_1_AR antibodies are functional, and the presence of these antibodies in a group of healthy individuals still needs to be clarified.^77^, ^80^ In the basic experiment, β_1_AR antibodies from some patients served as receptor‐sensitizing agents by increasing basal and agonist‐stimulated receptor activity, whereas those from other patients acted as partial agonists by decreasing agonist‐stimulated receptor activity.^80^, ^81^

## 6 GPCR SIGNALING AND S‐NITROSYLATION

Protein S‐nitrosylation is a covalent post‐translational modification of cysteines with nitric oxide (NO) to form protein S‐nitrosothiols (SNOs), providing a ubiquitous mechanism for cellular signaling.^82^, ^83^ The NO moiety is generally provided by NO produced from neuronal (nNOS/NOS1), inducible (iNOS/NOS2) or endothelial (eNOS/NOS3) nitric oxide synthase.^83^, ^84^ Numerous cellular functions, such as enzymatic activity, protein–protein interactions, and subcellular localization can be affected by protein S‐nitrosylation. While some proteins such as dynamin, ryanodine receptor, and βarr2 can be activated by physiological S‐nitrosylation, others like GRK2 and eNOS can be inhibited.^85^

Following GPCR activation, the resultant NO can mediate signaling through S‐nitrosylation and inhibit G protein coupling.^13^, ^85^ Interaction between GRK2 and eNOS leads to S‐nitrosylation of GRK2 at cysteine 340, inhibiting its kinase activity to prevent βAR desensitization. Reciprocally, eNOS phosphorylation and activity can be reduced by GRK2.^86^ Both isoforms (βarr1 and βarr2) can be nitrosylated at multiple loci by all three NOS isoforms, resulting in distinct signaling.^13^, ^87^ S‐nitrosylation of either βarr1 or βarr2 by n/iNOS inhibits recruitment to multiple GPCRs, providing a preventative mechanism against desensitization and internalization, whereas S‐nitrosylation of βarr2 by eNOS at Cys410 augments receptor internalization.^13^, ^87^, ^88^

## 7 GRKs AND ALDOSTERONE SIGNALING IN HF

Aldosterone exerts significant effects on the cardiovascular system, including vascular tone, cardiac contractility, and cardiac remodeling via the mineralocorticoid receptor (MR), which is responsible for all genomic effects of aldosterone.^89^, ^90^ MR plays an important role in promoting cardiac dysfunction and remodeling even in the absence of evident cardiac injury.^12^, ^91^, ^92^ Although aldosterone requires MR for its actions, it can also carry out some of its actions independent of MR, mostly through activation of the G protein‐coupled receptor 30 (GPR30) or G protein‐coupled estrogen receptor (GPER), a plasma membrane GPCR.^12^, ^93^ GPER takes part in many cardiovascular pathologies, providing favorable results in terms of cardiac function and structure.^94^, ^95^ GRK2 opposes antiapoptotic GPER signaling through phosphorylating and desensitizing GPER.^12^, ^22^

There is a bidirectional interaction between MR and GRKs. Experimental studies showed that MR promoted HF by activating GRK2‐dependent apoptosis and GRK5‐dependent hypertrophy.^90^, ^96^ Unlike GRK2, GRK5 is usually anchored to cell membrane phospholipids.^12^, ^97^ β_2_AR activates GRK5 by mobilizing intracellular calcium (Ca^2+^) via a non‐canonical cAMP‐independent signaling pathway.^12^, ^98^ Upon activation by β_2_AR, GRK5 translocates from the cell membrane to the cytoplasm where it phosphorylates MR and blocks the transcriptional activity of this receptor.^12^ β_1_AR does not stimulate GRK5 activation, which might be due to the inability of β_1_AR to activate Ca^2+^‐dependent signaling pathways.^12^ Interestingly, simultaneous stimulation of both β_1_AR and β_2_AR did not activate GRK5, which is likely because β_1_AR antagonizes the ability of β_2_AR to stimulate Ca^2+^‐dependent GRK5 cytoplasmic/nuclear translocation.^12^, ^99^

## 8 GRK2 IN HYPERTROPHIC AND DILATED CARDIAC MODELS

Cardiac remodeling refers to the structural and functional abnormalities of the heart that develop in response to various stimuli such as myocardial ischemia, arrhythmia, and pressure and volume overload. A broad range of cells are involved in the initiation and progression of cardiac remodeling.^100^ The changes in left ventricular (LV) volume, mass and function are progressive and without treatment ultimately end up in HF.^100^, ^101^

The therapeutic potential of GRK2 inhibition in HF was studied in mice with cardiac‐specific expression of a carboxyl‐terminal peptide of GRK2 (βARKct), a known GRK2 inhibitor.^7^ The βARKct peptide binds to Gβγ; therefore, Gβγ dissociated from GPCRs becomes scavenged, which prevents GRK2 from being recruited to the cell membrane.^23^ βARKct transgenic mice demonstrated attenuation of LV contractility in response to isoproterenol stimulation as well as reduced myocardial adenylyl cyclase activity and functional coupling of βARs.^102^ In a genetic model of murine‐dilated cardiomyopathy through knockout of the muscle LIM protein (MLP^−/−^), MLP^−/−^ mice mated with transgenic mice with cardiac‐targeted overexpression of βARKct showed less cardiac dilatation and better function.^103^ Similar results were observed in another dilated cardiomyopathy model utilizing βARKct mice mated with transgenic mice overexpressing the sarcoplasmic reticulum Ca^2+^‐binding protein, calsequestrin.^104^

GRK2 has also been involved in the pathophysiology of cardiac hypertrophy (Figure 2).^23^ In a rat myoblast cell line, GRK2 overexpression promoted hypertrophy by upregulation of NFκB activity in a phosphorylation‐dependent manner.^105^ Stimulation of isolated cardiac myocytes with angiotensin II and phenylephrine led to hypertrophy and enhanced GRK2 expression as a result of protein kinase B (PKB/Akt) phosphorylation and subsequent inactivation of glycogen synthase kinase 3 beta (GSK3β), resulting in enhanced NFAT activity.^106^ Of note, NFAT is a transcription factor for pro‐hypertrophic genes.^107^ In animal model of cardiac hypertrophy, the GRK2 inhibitor paroxetine attenuated adverse cardiac remodeling by inhibiting NFκB mediated prohypertrophic and profibrotic gene expression.^108^

TGF‐β1 promotes myocardial hypertrophy and fibrosis by increasing contractile protein synthesis in cardiomyocytes and extracellular matrix production by fibroblasts.^109^, ^110^, ^111^ Crosstalk between TGF‐β1 and GRK2 promotes cardiac hypertrophy and fibrosis.^112^ GRK2 overexpression can enhance TGF‐β1 activity and its downstream signaling in cardiomyocytes, contributing to cardiac hypertrophy.^112^ Conversely, TGF‐β1 is capable of inducing GRK2 expression in vascular smooth muscle cells, cardiomyocytes, and fibroblasts.^112^, ^113^, ^114^ Mice with Sjogren's syndrome further demonstrated the interaction between TGF‐β and GRK2.^115^ It was shown that GRK2 and Smad2/3 interacted in mouse salivary gland epithelial cells to positively regulate TGF‐β‐Smad signaling activation, resulting in a TGF‐β‐GRK2 positive feedback loop that contributes to gland fibrosis. By blocking Smad2/3 nuclear translocation, hemizygous deletion of GRK2 reduced TGF‐β‐induced collagen I synthesis in salivary gland epithelial cells in vitro and prevented gland fibrosis in mouse Sjogren's syndrome. A recent study revealed that prostaglandin E2 (PGE2) suppresses the TGF‐β_1_‐GRK2 interaction in cardiomyocytes and cardiac fibroblasts, thereby improving cardiac hypertrophy and fibrosis.^112^

A mouse model of hypertrophic cardiomyopathy crossbred with βARKct mice exhibited improved systolic function and exercise tolerance, along with decreased cardiac remodeling and hypertrophic gene expression.^116^ Hypertrophic gene expression can also be inhibited by the RGS domain of GRK2 as result of Gαq inhibition. Mice with cardiac‐specific expression of the RGS domain of GRK2 were subjected to pressure overload and exhibited less ventricular hypertrophy and related gene expression.^31^ Systemic administration of a small molecule Gβγ inhibitor, gallein, showed similar results as βARKct and the RGS domain.^117^ Mice with transgenic expression of the short N‐terminal domain of GRK2, βARKnt, exhibited baseline cardiac hypertrophy; however, their response to chronic pressure overload was proportional and adaptive, characterized by preserved LV structure, reduced interstitial fibrosis, and enhanced cell survival signaling. βARKnt mice had increased βAR membrane density, attributable to a compensatory increase in GRK2 levels, and demonstrated βAR downregulation upon challenge with isoproterenol.^30^

The RAF kinase inhibitor protein (RKIP) can intervene in the control of several signaling pathways due to its multifunctionality. The MAP kinase cascade Raf/MEK/ERK1/2, which controls a number of illnesses, and the βAR signaling to protein kinase A (PKA), especially in the heart, are two important signaling pathways regulated by RKIP.^118^, ^119^ RKIP is a dual‐function protein whose activity is influenced by its phosphorylation status. In its unphosphorylated state, RKIP serves as a suppressor of metastatic cancer progression by reducing MAPK signaling. In contrast, when phosphorylated, RKIP helps protect against HF by enhancing βAR/PKA signaling. However, a prolonged rise in βAR and PKA signaling can be harmful.^119^ Furthermore, RKIP has the ability to indirectly disrupt GPCRs, which are upstream Raf‐1 activators. Therefore, RKIP is liberated from Raf‐1 and binds to GRK2 upon being phosphorylated by protein kinase C (PKC). In addition to increasing GPCR activation, this interaction between phosphorylated RKIP and GRK2 also causes MAPK to become overactivated since Raf‐1 will no longer be blocked by RKIP, which in turn activates downstream targets.^118^, ^119^, ^120^ RKIP inhibits GRK2 by interacting with the N‐terminal domain of GRK2. By this interaction, non‐conical functions of GRK2 are not affected by RKIP.^9^ As an inhibitor of GRK2, RKIP attenuates βAR desensitization and augments βAR‐mediated contraction. Unlike inhibition of GRK2 by the cardioprotective βARKct, transgenic mice overexpressing RKIP showed elevated GRK2 transcript levels and developed HF. The upregulation of cardiac GRK2 transcript levels in these mice could be due to sensitization of βAR by RKIP.^9^ Moreover, by this specific mode of GRK2 inhibition, RKIP sensitizes the GPCRs to their substrates such as angiotensin II receptor type 1, promoting cardiac fibrosis and hypertrophy.^9^, ^10^, ^121^ Of note, RKIP can inhibit the pro‐survival RAF1‐MAPK pathway, which also contributes to the development of HF by promoting cardiomyocyte death and cardiotoxic lipid overload.^9^

In contrast to the previously mentioned evidence, some studies indicate that RKIP may have positive effects on the heart. Overexpressing RKIP in the hearts of transgenic mice (RKIP‐tg) led to sustained activation of βAR and PKA, resulting in faster cardiac contraction and relaxation. Unlike catecholamines, which activate βAR and can cause harmful effects like arrhythmia and cardiac remodeling, RKIP‐mediated hypercontractility was well‐tolerated up to 12–14 months of age. RKIP selectively activated two subtypes of βAR, enhancing heart function via β_1_AR and protecting against arrhythmia and remodeling via βAR.^119^, ^122^ In the setting of myocardial ischemic reperfusion injury, increased mRNA expression of RKIP in response to long‐term administration of sodium hydrosulfide was found to be associated with decreased mRNA expression of NF‐κB.^123^ Notably, NF‐κB is promoter of inflammation, exacerbating the heart response to ischemic injury.^124^

cAMP as a widespread second messenger, regulates a variety of physiological and psychological processes.^125^ The effects of cAMP are carried out by four main downstream effectors: PKA, cyclic nucleotide‐gated (CNG) ion channels, Popeye domain‐containing (POPDC) proteins, and exchange proteins directly activated by cAMP proteins (Epac).^125^ Epac proteins are present in different compartments of the cell including the nucleus and plasma membrane and manage separate cellular responses in a spatiotemporally regulated manner.^125^, ^126^ Epac has been shown to regulate many cAMP‐dependent cardiovascular functions, such as Ca^2+^ handling and vascular tone.^127^ GRK‐induced β1AR phosphorylation results in a conformational change in βarr allowing its interaction with Epac1.^1^ Activated Epac1 promotes activation of Ca^2+^ sensitive protein CaMKII, promoting phosphorylation of histone deacetylase 4 (HDAC4), and in turn relieving inhibition of the hypertrophic transcription factor, myocyte enhancer factor 2 (MEF2).^1^ Moreover, the interaction of Epac1 with βarr2 can induce a switch from β2AR non‐hypertrophic signaling to a β1AR‐like pro‐hypertrophic signaling cascade (Table 2).^1^, ^128^

**TABLE 2:** Effects of GRK2 Inhibition or Pharmacological Interventions on Hypertrophy and Cardiac Dilation.

Ref/Year	Animal/Method	Drug/Application	Result
24839449/2012	In mice with βARKct	Isoproterenol stimulation	↓ LV contractility
			↓ Myocardial adenylyl cyclase activity
			↓ Functional coupling of βARs
9618528/1998	In mice through knockout of muscle LIM protein (MLP−/−)	Mated with mice with overexpression of the βARKct	↓ Cardiac dilatation and better function
11331748/2001	In mice with βARKct	Mated with mice overexpressing calsequestrin	↓ Cardiac dilatation and better function
26224342/2015	In vitro, in rat myoblast cell line (H9C2)	‐	↑ Regulation of NFκB activity➔↑Hypertrophy
28759639/2017	In vitro, in cardiac myocytes	Angiotensin II and phenylephrine	Hypertrophy
			PKB phosphorylation and inactivation of GSK3β ➔ ↑ GRK2 expression
			↑ NFAT activity
15336966/2004	In mice with cardiac hypertrophy	Paroxetine	Inhibiting NFκB ➔ ↓ Adverse cardiac remodeling
35934102/2022	In vitro, in cardiomyocytes with GRK2 overexpression	‐	↑ TGF‐β1 activity
35934102/2022	In vitro, in cardiomyocytes and cardiac fibroblasts	PGE2	↓ Cardiac hypertrophy
			↓ Cardiac fibrosis
11306600/2001	In mice with hypertrophic cardiomyopathy	Crossbred with βARKct mice	↑ Systolic function
			↑ Exercise tolerance
			↓ Cardiac remodeling
			↓ Hypertrophic gene expression
27016525/2016	In mice with cardiac‐specific expression of the RGS domain of GRK2	Pressure overload	↓ Ventricular hypertrophy
			↓ Gene expression related to ventricular hypertrophy
24703913/2014	In mice subjected to transverse aortic constriction (TAC)	Gallein	↓ Ventricular hypertrophy
			↓ Gene expression related to ventricular hypertrophy
33548241/2021	In mice with βARKnt	Chronic pressure overload	↔ LV structure
			↓ Interstitial fibrosis
			↑ Cell survival signaling
33548241/2021	In mice with βARKnt	Isoproterenol	↓ Regulation of βAR
35203304/2022	In Tg‐RKIP mice with RKIP overexpression	‐	↑ Regulation of cardiac GRK2 transcript levels
			↓ βAR desensitization
			↓ βAR‐mediated contraction
			Inhibits pro‐survival RAF1‐MAPK pathway ➔ Cardiomyocyte death and cardiotoxic lipid overload➔ development of HF

## 9 GRK2 IN ISCHEMIC HEART MODELS

Ischemic myocardial injury is one of the leading causes of LV dysfunction, which is a major determinant of clinical outcomes.^129^, ^130^ Preserving myocardial tissue from damage caused by ischemic injury has emerged as a crucial objective in medical treatment.^129^, ^131^ A substantial body of evidence indicates that the level of GRK2 is upregulated acutely after myocardial ischemic injury in both clinical and pre‐clinical models, and this upregulation is strongly linked to disease severity and the progression to HF.^132^ In an ischemia/reperfusion (I/R) model, while cardiac‐specific GRK2‐overexpressing transgenic mice had greater infarct size, βARKct‐overexpressing mice showed smaller damage relative to the control group. Increased Akt activity and NO production were reported to be the underlying mechanism of GRK2 inhibition with the βARKct.^133^ Cardiomyocyte‐specific GRK2 ablation of mice either at birth or prior to I/R injury exerted smaller infarct size and improved cardiac function. Attenuated myocyte apoptosis in ablated mice was attributed at least partially to Akt/Bcl‐2 mediated mitochondrial protection.^134^

In an isolated rat heart I/R model, GRK2 protein levels were found markedly decreased during the ischemic and early phase of reperfusion. Further investigation suggested that boosted GRK2 phosphorylation at Ser670 during ischemia promoted GRK2 degradation by the proteasome, whereas phosphorylation at Ser685 in early reperfusion favored calpain‐mediated GRK2 proteolysis.^21^ Concurrently, degradation of Akt protein and prolyl isomerase Pin1 (Akt stabilizing factor) with GRK2 suggested a potential functional link between these players during I/R injury. Combined administration of proteasome and calpain inhibitors prevented early GRK2/Pin1/Akt degradation and attenuated I/R myocardial injury.^21^ Of note, Akt is a component of the reperfusion injury salvage kinase pathway (RISK) and has an important role in cardioprotection.^21^, ^135^, ^136^

Cardiac fibroblasts are instrumental in the homeostasis of the myocardial extracellular matrix. Upon cardiac injury, the transformation of fibroblast to an activated myofibroblast state plays a part in the repair and remodeling process of the heart.^137^ Fibroblast GRK2 knockout mice exhibited smaller infarct size and better cardiac function post‐I/R injury, accompanied by reduced fibrosis and fibrotic gene expression. Notably, these favorable effects were accompanied by diminished neutrophil infiltration and tumor necrosis factor‐α (TNF‐α) expression.^114^ Similarly, inhibition of the Gβγ‐GRK2 axis limited pathological myofibroblast activation and interstitial fibrosis in the I/R model of mice.^137^

In a mouse model of myocardial infarction (MI), GRK2 ablation after birth demonstrated attenuated adverse LV remodeling and preserved βAR responsiveness post‐injury. Furthermore, GRK2 ablation conducted 10 days after MI improved the survival rate, preserved myocardial contraction, and halted adverse remodeling.^138^ Wild‐type mice treated for 4 weeks with paroxetine, a selective serotonin reuptake inhibitor characterized by GRK2 inhibitory action, starting at 2 weeks after MI showed better results in terms of cardiac structure and function alongside several hallmarks of HF. Interestingly, the beneficial effects of paroxetine were obviously greater than those that received β‐blocker therapy, a cornerstone therapy of HF.^16^, ^139^ Unlike in animals, paroxetine failed to improve LV remodeling in patients experiencing MI. The beneficial effects of paroxetine might be masked in patients due to other pharmacological treatments that are already proved to improve cardiac remodeling.^140^

Intracoronary adenoviral‐mediated gene delivery of a peptide inhibitor of betaARK1 (βARKct) to a rabbit model of MI revealed improved cardiac function.^141^, ^142^ Rabbits that received adenoviral‐βARKct and were subjected to prolonged cardioplegic arrest exerted better LV function.^143^ Long‐term effects of inhibition of betaARK1 via gene delivery on HF have also been tested in experimental studies. Using stable myocardial gene delivery of βARKct via adeno‐associated virus serotype 6 (AAV6) to a porcine model of HF after MI improved the echocardiographic and hemodynamics results 6 weeks after gene transfer.^144^ Similarly, in rat HF model after MI, gene delivery with AAV6 resulted in enhanced cardiac contractility and reversed LV remodeling at least 12 weeks after delivery.^145^

Although elevated GRK2 expression has worse outcomes in many cardiovascular diseases, reduced GRK2 expression may also be associated with detrimental results. Within 24 hours post‐MI, selective GRK2 activity suppression was detected in the arrhythmogenic subepicardial border zone tissue overlying the infarct, which sensitizes the animals to beta‐adrenergic stimulation and malignant tachyarrhythmias.^146^, ^147^, ^148^

Neovascularization of the ischemic area is essential for post‐infarct cardiac remodeling.^149^ GRK2 plays an important role in vascular development. Mouse embryos with systemic or endothelium‐selective GRK2 ablation developed vascular malformations.^150^ GRK2 expression levels and localization in the endothelial cell are necessary for the proper functioning of the endothelium.^151^, ^152^ An imbalance and abnormal activity of GRK2 may influence cell proliferation, migration, and other behaviors.^152^ Chemical inhibition of GRK2 reduced endothelial dysfunction in type 2 diabetic mice by improving βarr2 translocation and ameliorating Akt/eNOS signal dysfunction (Table 3).^153^, ^154^

**TABLE 3:** Role of GRK2 and effects of drug or pharmacological intervention on ischemic hearts.

Ref/Year	Animal/Method	Drug/Application	Result
20814022/2011	In mice subjected to I/R and overexpressing GRK2	‐	↑ Infarct size
	In mice with βARKct		Relatively smaller infarct size
23805205/2013	In mice either at birth or prior to I/R injury	GRK2 ablation	Smaller infarct size
			↑ Cardiac function
			↓ Myocyte apoptosis
31594751/2019	In isolated rat I/R model	GRK2 phosphorylation at Ser670 during ischemia	↓ GRK2 protein levels
		Phosphorylation at Ser685 in early reperfusion	
31594751/2019	In isolated rat hearts of I/R‐myocardial injury	Combined administration of proteasome and calpain inhibitors	Prevented GRK2 degradation
			↓ I/R myocardial injury
27601479/2017	Fibroblast GRK2 knockout mice	‐	Smaller infarct size
			↑ Cardiac function post‐I/R injury
			↓ Fibrosis
			↓ Fibrotic gene expression
			↓ Neutrophil infiltration and tumor necrosis factor‐α expression
28818206/2018	In mice post‐I/R	GRK2 ablation and Gβγ‐	↓ Pathological myofibroblast activation
		GRK2 inhibition	↓ Interstitial fibrosis
18635825/2009	In mice subjected to MI	GRK2 ablation after birth	↓ Adverse LV remodeling
		GRK2 ablation conducted 10 days after MI	↔ βAR responsiveness
			↑ Survival rate
			↔ Myocardial contraction
			Halted adverse remodeling
25739765/2016	In wild‐type mice	Paroxetine	↑ Cardiac structure and function
35898267/2022	In patients experiencing MI	Paroxetine	↔ LV remodeling
10779554/2000	In rabbits subjected to MI	Intracoronary adenoviral‐mediated gene delivery of βARKct	↑ Cardiac function
11238278/2001			↑ LV function
22261894/2013	Porcine model of HF after MI	Myocardial gene delivery of βARKct via AAV6	↑ Echocardiographic results
			↑ Hemodynamics results
19103992/2009	In rats subjected to MI	Gene delivery of βARKct with AAV6	↑ Cardiac contractility
			↓ LV remodeling
28814745/2017	In mice with type 2 diabetes	Chemical inhibition of GRK2 (GRK2 siRNA or Methyl 5‐[2‐(5‐nitro‐2‐furyl)vinyl]‐2‐furoate)	↓ Endothelial dysfunction
22581458/2012			

## 10 GRK2 AND MITOCHONDRIAL FUNCTION IN HF

Myocardial tissue is energy deprived in HF and mitochondrial dysfunction is a driving force behind the energy supply–demand imbalance in the failing heart. Structural and functional abnormalities in the mitochondria of the failing heart lead to reduced ATP synthesis and excessive reactive oxygen species (ROS) formation, contributing to the worsening of the HF state.^155^, ^156^ In fibroblast, GRK2 overexpression enhanced mitochondrial biogenesis and ATP production. In the I/R mouse model of the limb, GRK2 levels in mitochondria transiently increased during ischemic conditions and then returned to basal level after reperfusion. Acute accumulation of GRK2 in mitochondria antagonized ATP loss after I/R. In vivo, GRK2 removal from skeletal muscle led to diminished ATP production and impaired tolerance to ischemia.^157^ In another study, inflammation induction via lipopolysaccharide increased mitochondrial GRK2 accumulation and biogenesis resulting in reduced ROS production and cytokine expression in macrophages.^158^

Acute and transient ionizing radiation exposure translocated GRK2 from the plasma membrane to mitochondria. Upon ionizing radiation exposure, mitochondrial damage occurs, characterized by alterations in mass, morphology, and respiration. While GRK2 overexpression exerted protective effects, its removal provoked mitochondrial damage. Another novelty of this experiment was the finding of a new interactome, MFN‐1 and 2 (Mitofusin‐1 and 2), that are bonded and phosphorylated by GRK2 after its interaction with HSP90. Of note, MFN‐1 and 2 are key regulators of mitochondrial fusion and recovery.^159^

Unlike the abovementioned studies, some authors have reported that GRK2 accumulation in mitochondria following stress is detrimental. Using in vivo mouse models of ischemic injury and cultured myocytes, mitochondrial localization of GRK2 was enhanced after ischemic and oxidative stress. It was proposed that phosphorylation of GRK2 at residue Ser670 within the carboxyl‐terminus by extracellular signal‐regulated kinases enhances GRK2 binding to HSP90, which chaperones the kinase to mitochondria. Mitochondrial accumulation of GRK2 after ischemic injury promoted pro‐death signaling and also led to increased Ca^2+^‐induced opening of the mitochondrial permeability transition pore.^160^ In mice with a S670A knock‐in mutation, GRK2 could not bind to HSP90 and translocate to mitochondria after IR injury. Mice with a S670A knock‐in mutation in endogenous GRK2 had smaller infarct size and better cardiac function post‐IR injury.^11^ It was proposed that there might be two pools of mitochondrial GRK2: a basal pool of unphosphorylated GRK2 that has a different role than the post‐stress, and Ser670 phosphorylated GRK2 that promotes cell death. Further in vitro investigation showed improved glucose‐mediated oxidation post‐IR injury in S670A knock‐in myocytes that was partially attributed to the maintenance of pyruvate dehydrogenase activity.^11^

Sub‐fractionation of purified cardiac mitochondria showed that GRK2 is already localized in multiple compartments of mitochondria independent of cardiac injury. GRK2 overexpression in mouse cardiomyocytes impaired fatty acid (FA) oxidation and increased superoxide levels. Conversely, GRK2 inhibition improved oxygen consumption rates and ATP production.^161^ In line with these findings, mice with cardiac‐specific overexpression of GRK2 reduced FA uptake and oxidation.^162^ Adenoviral‐mediated overexpression of GRK2 enhanced mitochondrial oxidative stress and ROS production, reportedly NOX4 mediated, alongside apoptosis. Adenoviral‐mediated expression of a GRK2 inhibitor attenuated ROS production and apoptosis in response to a beta‐agonist.^163^

Different experiments with different techniques have reported contradicting results regarding GRK2 activity in mitochondria.^164^ Apparently, timing is a consideration. Indeed, transient and acute accumulation of GRK2 in mitochondria might be protective in response to acute insults; however, chronic GRK2 hyperactivation might have a detrimental effect on mitochondrial regulation.^4^ The role of GRK2 in mitochondria regardless of physiological and pathological conditions requires more investigations (Table 4).

**TABLE 4:** Role of GRK2 and effects of pharmacological interventions on mitochondrial function in heart failure.

Ref/Year	Animal/Method	Drug/Application	Result
21983013/2012	In fibroblasts with GRK2 overexpression	‐	↑ Mitochondrial biogenesis
	In I/R mouse model of the limb	‐	↑ ATP production
	In vivo model of muscle ischemia	GRK2 removal from skeletal muscle	↑ GRK2 levels in mitochondria and returned to basal level after reperfusion
			↓ ATP production Impaired tolerance to ischemia
24036448/2014	In vitro, in macrophages with lipopolysaccharide‐induced inflammation	‐	↑ Mitochondrial GRK2 accumulation
			↓ ROS production and cytokine expression
29531822/2018	In vitro, exposed to ionizing radiation	‐	Mitochondrial damage
30538174/2018	In mice with a S670A knock‐in mutation GRK2 and IR injury		Smaller infarct size
	In vitro, S670A knock‐in myocytes post‐IR		↑ Cardiac function
			↑ Glucose‐mediated oxidation
26506135/2015	In mouse‐isolated cardiomyocytes	Adenoviral delivery of GRK2	↓ FA oxidation and ↑ superoxide levels
	In mice	GRK2 inhibition	↑ Oxygen consumption rates
			↑ ATP production
30171848/2018	In mice with GRK2 overexpression	‐	↓ FA uptake and oxidation
16762799/2006	In patients with heart failure	Mechanical unloading of the heart with a LV assist device	↓ Cardiac remodeling accompanied
			↑ βAR responsiveness
			↓ Lymphocyte GRK2 levels
20443948/2010	In patients with heart failure	Underwent heart transplantation	↓ Blood GRK2 levels
23689525/2020	In patients with advanced HF	Exercise training	↓ Lymphocyte GRK2 protein levels
			Better prognosis
			↑ Insulin resistance
			↓ FA oxidation
			↑ Cardiomyocyte oxidative stress
31680450/2020	In diabetic patients with LV diastolic dysfunction	–	↑ Insulin resistance
	In diabetic mice, early‐stage cardiomyopathy	–	↓ FA oxidation
			↑ Cardiomyocyte oxidative stress
20335112/2010	In vitro, in cardiomyoblast culture	High glucose medium	↑ GRK2‐mRNA levels

## 11 GRK2 IN CIRCULATING LYMPHOCYTES IN HF

Increased GRK2 levels in circulating lymphocytes reflect the sustained hyperactivation of βAR because of exposure to high catecholamine levels in HF, suggesting a more reliable marker of adrenergic nervous system hyperactivity than circulating NE levels.^14^, ^165^ In myocardial biopsies from explanted failing human hearts, a direct correlation between myocardial and lymphocyte GRK2 activities was detected. Elevated lymphocyte GRK2 activity in parallel with that of the myocardium was associated with the loss of βAR responsiveness and enhanced peripheral NE circulating levels.^166^ These findings were supported by mechanical unloading of the heart with a LV assist device, leading to reverse cardiac remodeling accompanied by restoration of βAR responsiveness and decreased lymphocyte GRK2 levels (Figure 2).^167^ Similarly, blood GRK2 levels significantly dropped in patients who underwent heart transplantation.^168^ Exercise training also has been shown to reduce lymphocyte GRK2 protein levels, which was strongly associated with better prognosis in advanced HF.^165^ The prognostic value of blood GRK2 levels has also been confirmed in a larger HF population, which reported that GRK2 levels exhibited additional independent prognostic and clinical information over demographic and clinical variables.^169^ Not only in chronic HF but also in acute coronary syndrome lymphocyte GRK2 levels were able to predict the future of cardiac function. Increased lymphocyte GRK2 levels during acute myocardial infarction were associated with worse cardiac function.^170^

Besides HF, high lymphocyte GRK2 levels were found in diabetic patients with LV diastolic dysfunction. Increased GRK2 expression in the myocardial tissue of diabetic mice in early‐stage diabetic cardiomyopathy suggests involvement of this kinase in the development of the pathology.^171^ In vitro investigation revealed that the GRK2‐mRNA levels increased in cardiomyoblast cultured with high glucose medium, suggesting induction of GRK2 with hyperglycemia.^172^ GRK2 might be involved in diabetic cardiomyopathy in many ways other than βAR‐mediated signaling, including insulin resistance, FA oxidation, and cardiomyocyte oxidative stress.^171^

## 12 GRK2 AND LIPID METABOLISM IN HF

The heart is capable of using a variety of substrates such as FAs, lactate, glucose, ketone bodies, and amino acids to produce energy. In physiologic conditions, up to 60% of ATP production is derived from FA metabolism.^173^ While some of the FAs taken into the cell are burned for energy in mitochondria, another part is used for triacylglycerides (TAG) synthesis in the smooth endoplasmic reticulum (ER) and stored in lipid droplets for energy and the structure of the cell membrane.^173^ Alterations in metabolism during pathologic conditions are an important contributor to adverse LV remodeling.^174^ During HF, the heart can switch its substrate preference from FAs to glucose, although FA oxidation remains the most important source of energy production.^175^ A mismatch between lipid uptake and lipid utilization can cause intracellular lipid accumulation, mainly of triglycerides (TGs), diacylglycerols (DAGs), and ceramides, as well as cholesterol and its derivatives.^175^ Ceramides and DAGs act as lipotoxic mediators and contribute to cardiomyocyte death via several mechanisms including insulin resistance, inflammation, ROS generation, and ER and mitochondrial stress.^175^, ^176^ Accumulation of free cholesterol within cellular membranes increases both cell and organelle rigidity, contributing to cardiomyocyte cytotoxicity by altering membrane permeability and mitochondrial dynamism.^175^, ^177^

GRK2 can regulate both white and brown adipose tissues function and architecture, effecting whole‐body FA metabolism and energy expenditure.^29^, ^178^ Using an inducible mouse knockout model, GRK2 ablation after high‐fat diet (HFD)‐dependent obesity and insulin resistance improved insulin sensitivity and whole‐body glucose homeostasis. Moreover, these animals were characterized by reduced fat mass and smaller adipocytes despite continued HFD.^179^ On the other hand, a study with cardiac‐specific GRK2 overexpressing mice showed accelerated cell death when isolated cardiomyocytes were cultured with palmitate, suggesting an impairment in FA metabolism. The upregulation of GRK2 was proposed to reduce FA‐specific catabolic pathways and impair metabolic adaptation of mitochondria in pathologic conditions (Figure 2).^180^

In another diet‐induced obesity model, cardiac‐restricted expression of an amino‐terminal peptide of GRK2 (βARKnt: competitive inhibitor of GRK2) attenuated adverse cardiac remodeling through direct modulation of insulin signaling pathways within cardiomyocytes during metabolic stress. Furthermore, improved metabolic flexibility and energy utilization were accompanied by protected maladaptive visceral adipocyte hypertrophy, and induced visceral fat browning.^181^ In agreement with these findings, hemizygous‐GRK2 mice were observed to be protected from HFD‐promoted intramyocardial lipid accumulation, cardiomyocyte hypertrophy, and fibrosis. Underlying protective mechanisms might be preserved PPARα and peroxisome proliferator‐activated receptor γ‐PPAR γ‐coactivator proteins (PGC1) levels in hemizygous mice.^182^ Of note, PPARα cooperates with PGC1 to upregulate genes implicated in FA import and β‐oxidation in the mitochondria.^182^

Unlike the abovementioned studies, transgenic mice with cardiac‐specific expression of a peptide inhibitor of GRK2 (TgβARKct) were found to be more susceptible to HFD‐induced obesity. Conversely, mice with cardiac‐specific overexpression of GRK2 (TgGRK2) had resistance to HFD‐induced obesity.^183^ GRK2 signaling not only altered myocardial branched‐chain amino acid (BCAA) and endocannabinoid metabolism but also circulating metabolite profiles of these. These results suggest that metabolic control of GRK2 signaling in mice fed a HFD goes beyond the heart and controls whole‐body metabolism. The discrepancy between the studies was attributed to the possibility that the effects of cardiac‐specific GRK2 downregulation might be masked by systemic downregulation of GRK2 owing to its direct effects on the other tissue, mainly adipose tissue.^183^

Adipose tissue is an endocrine organ secreting many endocrine factors including adiponectin. Adiponectin acts through AdipoR1 and R2 receptors, exerting anti‐inflammatory and anti‐apoptotic activity alongside energy homeostasis in many organ systems.^184^ Some researchers determined that GRK2 can phosphorylate and consequently desensitize AdipoR1 in cardiomyocytes of the failing heart, abating AdipoR1‐mediated cytoprotective actions.^185^ Phosphorylation of AdipoR1 at Ser205 by GRK2 resulted in clathrin‐dependent endocytosis and then lysosomal‐mediated degradation, responsible for AdipoR1 desensitization (Table 5).^186^

**TABLE 5:** Diverse roles of GRK2 and Effects of pharmacological interventions on lipid metabolism in heart failure.

Ref/Year	Animal/Method	Drug/Application	Result
26198359/2015	In mouse model for post‐HFD obesity	GRK2 ablation	↑ Insulin sensitivity and whole‐body glucose homeostasis
			↓ Fat mass and smaller adipocytes
35269919/2022	In cardiomyocytes from mice with GRK2 overexpression	Cultured in palmitate	↓ FA metabolism
35818501/2022	In diet‐induced obesity model with βARKnt	‐	↓ Adverse cardiac remodeling
			↑ Metabolic flexibility and energy utilization
27832814/2016	In hemizygous‐GRK2 mice	HFD	Protected from intramyocardial lipid accumulation, cardiomyocyte hypertrophy and fibrosis
25696921/2016	In cardiomyocytes from heart failure	Phosphorylation of AdipoR1 at Ser205 by GRK2	Clathrin‐dependent endocytosis
35611695/2023			Lysosomal‐mediated degradation
			AdipoR1 desensitization

## 13 GRK2 AND INSULIN SIGNALING IN HF

Systemic insulin resistance is a predisposing factor for developing HF independent of MI, hypertension, and hyperlipidemia. On the other hand, HF itself worsens systemic insulin resistance. Furthermore, the heart itself becomes resistant to insulin in terms of insulin‐mediated glucose uptake and glucose oxidation.^187^ Lower glucose utilization and oxidative reduction due to insulin resistance lead to an imbalance between the uptake and oxidation of FAs, resulting in mitochondrial dysfunction.^188^ Oxidative stress, inflammation, and impaired energy metabolism as a result of insulin resistance promote the development of cardiac fibrosis, hypertrophy, and dysfunction.^176^, ^187^, ^188^

Chromic βAR stimulation and GRK2 upregulation, both of which are seen in HF, are involved in the development of insulin resistance. Cells overexpressing βAR cause GRK2 accumulation in the cell membrane, resulting in IRS1 inactivation and reduced glucose uptake in response to insulin.^20^, ^189^, ^190^ GRK2 inhibition was shown to normalize fasting glycemia and improve glucose tolerance.^20^, ^179^ Some authors reported a physical interaction between GRK2 and the insulin receptor in the heart.^191^ Insulin promotes the recruitment of GRK2 to β2AR (for which IRS2 is necessary) and subsequently β_2_AR phosphorylation and internalization.^191^ β2AR phosphorylation can switch receptor coupling from Gαs to Gαi, resulting in inhibition of βAR‐activated AC‐cAMP‐PKA signaling pathway and depressed cardiac contraction.^191^, ^192^, ^193^

Positron emission tomography studies with transgenic mice revealed that cardiac‐specific overexpression of GRK2 desensitizes insulin signaling by phosphorylating IRS1 and consequently inhibiting membrane translocation of the glucose transporter GLUT4, resulting in glucose uptake inhibition, especially after ischemic injury.^194^ Conversely, GRK2 inhibition improved insulin signaling and normalized glucose uptake. Inhibition of GRK2 by KRX‐C7 peptide in an animal model of type 2 diabetes ameliorated the pathologic mechanism underlying diabetic cardiomyopathy, including inflammatory and cytokine responses, oxidative stress, and patterns of fetal gene expression alongside insulin sensitivity and glucose homeostasis.^195^

Interestingly, a recent study discovered the surprising fact that in both lean and obese mice, hepatic GRK2 impairment has no influence on insulin resistance, glucose homeostasis, or other critical metabolic parameters.^196^ It was shown that there was no significant difference in glucose tolerance, insulin sensitivity, in vivo gluconeogenesis, or glucagon‐induced hyperglycemia using hepatocyte‐specific GRK2 knockout mice. Similarly, the absence of hepatocyte GRK2 had no effect on plasma levels of insulin, glucagon, free fatty acids, or ketone bodies. The results of this investigation imply that other metabolically significant organs and cell types are probably involved in the modifications in insulin resistance brought on by changed GRK2 activity.^29^, ^189^, ^196^

GRK2 exists as a player downstream of the aldosterone signaling pathway. Using 3 T3 cells, aldosterone upregulated GRK2, weakened insulin signaling, enhanced the negative phosphorylation of IRS1, and reduced Akt activity. The GRK2 inhibitor, CMPD101, noticeably dampened the effects of aldosterone and prevented both insulin and βAR signaling dysfunction. These findings were confirmed in an in‐vivo model of hyperaldosteronism with cardiac‐specific GRK2‐knockout mice (Table 6).^28^

**TABLE 6:** Roles of GRK2 and effects of drugs and pharmacological interventions on insulin signaling in heart failure.

Ref/Year	Animal/Method	Drug/Application	Result
29166798/2018	In vitro, in cells overexpressing βAR and GRK2 accumulation in cell membrane	–	IRS1 inactivation
33467677/2021			↓ Reduced glucose uptake in response to insulin
19620130/2009			
26198359/2015	In vitro, in cells overexpressing βAR and GRK2 accumulation in cell membrane	GRK2 inhibition	Normalize fasting glycemia
			Improves glucose tolerance
21518983/2011	In mice with ischemic injury with GRk2 overexpression	–	Desensitizes insulin
			Inhibits glucose uptake
30934608/2019	In animal model of type 2 diabetes	KRX‐C7	↓ Cardiomyopathy
			↓ Inflammatory and cytokine responses
			↓ Oxidative stress
			↑ Insulin sensitivity
			↑ Glucose homeostasis
31447681/2019	In vitro, in 3 T3 cells	Aldosterone	↑ GRK2
			↓ Insulin signaling
			↑ Negative phosphorylation of IRS1
			↓ Akt activity
31447681/2019	In vivo model of hyperaldosteronism with GRK2‐knockout mice	CMPD101	↓ Effects of aldosterone
			Prevented insulin and βAR signaling dysfunction

## 14 CONCLUSIONS AND FUTURE DIRECTIONS

GPCRs are the largest family of transmembrane proteins, with βARs being a well‐known sub‐type responsible for a variety of functions in the heart. GRKs and βarr are two essential elements that carry out GPCR signaling. GRK2 is abundant in the heart, functioning in a wide range of critical signaling under physiological or pathological conditions. Overexpression of GRK2 is associated with various cardiovascular pathologies (Figure 2). This kinase has been experimentally targeted in several cardiovascular pathologies such as hypertension and hypertrophic cardiomyopathy, metabolic syndrome, type 2 diabetes, and nonalcoholic fatty liver disease. The vast majority of preclinical studies have shown that inhibition or deletion of GRK2, whether before or after induction of cardiac injury, is protective. The actions of GRK2 are not limited to canonical GPCR signaling, as it can phosphorylate many non‐GPCR substrates.

Sympathetic nervous system hyperactivity in HF leads to increased GRK2 levels in the heart. Initially, augmented GRK2 levels appear to be beneficial to myocardial tissue by counterbalancing beta‐adrenergic overdrive. However, persistent GRK2 overactivity becomes maladaptive, worsening HF progression through GPCR desensitization and downregulation, insulin resistance, mitochondrial dysfunction and apoptosis. The adrenal gland is an important source of catecholamines, with GRK2 taking part in the regulation of epinephrine secretion. In HF, GRK2 levels increase in the adrenal gland, making it a significant target alongside myocardial tissue for HF treatment.

**FIGURE 3:**
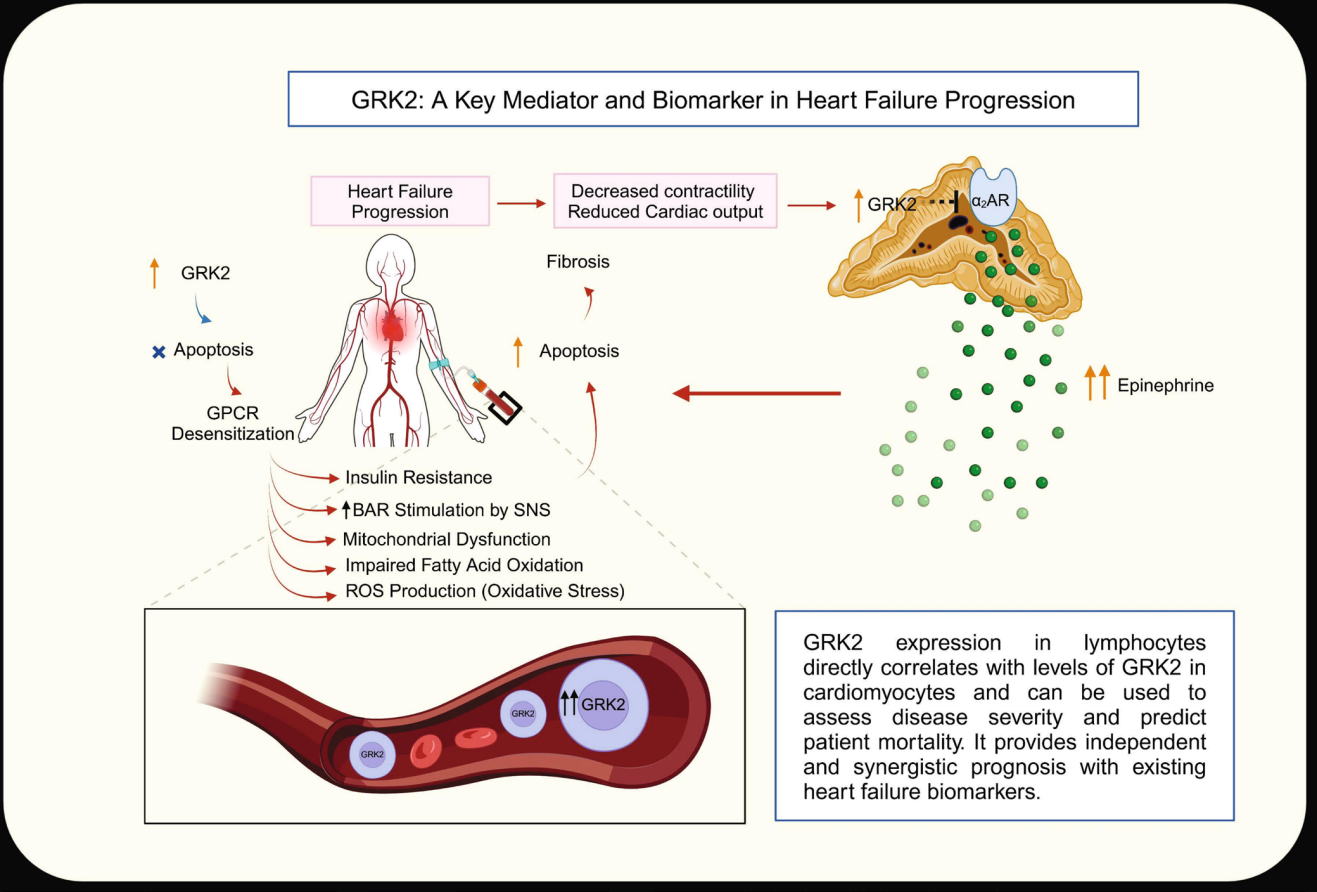
GRK2: A Key Mediator and Biomarker in Heart Failure Progression. In the early stages of heart failure, elevated GRK2 expression does not directly induce apoptosis. However, as heart failure advances, increased GRK2 expression contributes to various pathological effects, including GPCR desensitization, insulin resistance, heightened βAR stimulation by the SNS, mitochondrial dysfunction, impaired fatty acid oxidation, and ROS production. These processes collectively lead to apoptosis and cardiac fibrosis. Concurrently, heart failure is characterized by reduced myocardial contractility and diminished cardiac output. Elevated GRK2 further exacerbates these effects by inhibiting α2ARs, which regulate catecholamine secretion. This inhibition results in increased epinephrine release, driving cardiac remodeling, apoptosis, and fibrosis, thereby perpetuating the progression of heart failure. GRK2, G‐protein‐coupled receptor kinase 2; GPCR, G‐protein‐coupled receptor; βAR, β‐adrenergic receptor; SNS, sympathetic nervous system; ROS, reactive oxygen species; α2Ars, α2‐adrenergic receptors.

Like myocardial tissue, sustained hyperactivation of βAR due to high catecholamine level exposure in HF, increases GRK2 levels in circulating lymphocytes. GRK2 levels in lymphocytes seem to be a reliable non‐invasive method for monitoring HF status and guiding treatment. Given the fact that immune cells take part in every stage of HF, further investigation is required to explain the importance of GRK2 in lymphocytes beyond its potential as a follow‐up marker for HF (Figure 3).

The multidomain structure of GRK2 renders it multifunctional. Moreover, GRK2 expression levels, activity, and location within the cell contribute to its functional diversity. The RGS domain can inhibit hypertrophic gene expression, while the N‐terminal domain protects the heart against chronic pressure overload and improves energy metabolism by modulating insulin signaling. Cardiac‐specific expression of a carboxyl‐terminal peptide of GRK2 (βARKct), a known GRK2 inhibitor, attenuates adverse cardiac remodeling in hypertrophic, dilatated and ischemic heart models.

Fibrosis is a key component of cardiac remodeling. GRK2 can enhance cardiac fibrosis by increasing TGF‐β1 activity. Deleting GRK2 in cardiac fibroblasts reduces fibrosis. Neovascularization plays a crucial role in cardiac remodeling and GRK2 expression levels in endothelial cells are necessary for proper endothelial function. Selective GRK2 ablation in endothelial cells can lead to vascular malformations. Conversely, GRK2 inhibition improves endothelial function in type 2 diabetic mice. There is limited research on the role of GRK2 in neovascularization, especially after ischemic injury.

Mitochondria are involved in HF pathogenesis in many ways. GRK2 in mitochondria is implicated in mitochondrial biogenesis, FA oxidation, ROS production, mitochondrial fusion, and apoptosis. While most studies favor GRK2 inhibition for improvement in mitochondrial function, conflicting results necessitate further investigation. FAs are inevitable sources of energy for myocardial tissue, and local and systemic metabolism can be regulated by GRK2. GRK2 accumulation in the cell membrane due to βAR overstimulation leads to insulin resistance, that is another component of HF pathogenesis. GPCR signaling modulation, including G protein coupling, GRK2, and β‐arrestin activity via S‐nitrosylation, presents another intriguing aspect of HF pathogenesis (Figure 3).

In conclusion, given its multifunctionality, GRK2 contributes to various mechanisms underlying HF pathogenesis. By targeting this kinase, the progression to HF can be slowed on multiple fronts simultaneously. However, there is still much to uncover regarding the complete involvement of GRK2 in HF.

## AUTHOR CONTRIBUTIONS

**Abdullah Kaplan:** Conceptualization; writing – original draft; writing – review and editing; supervision; resources; software; validation. **Lana El‐Samadi:** Software; writing – original draft; writing – review and editing; validation. **Rana Zahreddine:** Software; validation; writing – original draft; writing – review and editing. **Ghadir Amin:** Writing – review and editing; validation; supervision. **George W. Booz:** Writing – original draft; writing – review and editing; supervision; validation. **Fouad A. Zouein:** Conceptualization; visualization; writing – original draft; writing – review and editing; supervision; funding acquisition; validation.

## FUNDING INFORMATION

This work was supported by grants from the American University of Beirut Faculty of Medicine [grants number: MPP—320145; URB—103949; URB—104262; URB—104115] to FAZ. GWB was supported in part by the National Institute of General Medical Sciences of the National Institutes of Health under Award Number P20GM121334. The content is solely the responsibility of the authors and does not necessarily represent the official views of the National Institutes of Health.

## CONFLICT OF INTEREST STATEMENT

The authors have no conflict of interest to declare.

## Data Availability

Data sharing not applicable to this article as no datasets were generated or analysed during the current study.
